# A Functional Variant rs3093023 in *CCR6* Is Associated With IgA Nephropathy by Regulating Th17 Cells in a North Han Chinese Population

**DOI:** 10.3389/fimmu.2021.600598

**Published:** 2021-02-25

**Authors:** Yue-miao Zhang, Xing-zi Liu, Xu-jie Zhou, Li-jun Liu, Su-fang Shi, Ping Hou, Ji-cheng Lv, Hong Zhang

**Affiliations:** ^1^Renal Division, Department of Medicine, Peking University First Hospital, Beijing, China; ^2^Key Laboratory of Renal Disease, Ministry of Health of China, Beijing, China; ^3^Key Laboratory of Chronic Kidney Disease Prevention and Treatment (Peking University), Ministry of Education of China, Beijing, China

**Keywords:** IgA nephropathy, CCR6, Th17 cells, genetic association, functional annotation

## Abstract

C-C chemokine receptor 6 (*CCR6*) is a susceptibility gene of various immune-related diseases, which was suggested to be shared with immunoglobulin A nephropathy (IgAN). In this study, we aimed to identify the functional variants. First, we analyzed the associations of *CCR6* common and rare variants detected by multi-platform chips with IgAN susceptibility using imputation and identified 68 significantly associated common variants located in the regulatory region. Among them, rs3093023 showed both statistical significance (rs3093023-A, odds ratio [OR] = 1.15, *P* = 2.00 × 10^−2^) and the expression quantitative trait loci (eQTL) effect (*P* = 1.45 × 10^−3^). It was independently replicated (rs3093023-A, OR = 1.18, *P* = 5.56 × 10^−3^) and the association was reinforced in the meta-analysis (rs3093023-A, OR = 1.17, *P* = 6.14 × 10^−7^). Although rs3093023 was in a strong linkage disequilibrium with the reported *CCR6* functional variant dinucleotide polymorphism, *CCR6DNP*, the alleles of rs3093023 (G>A) rather than of *CCR6DNP* were shown differential nuclear protein binding effect by electrophoretic mobility shift assay. The RegulomeDB and JASPAR databases predicted Pou2f1 as the potential transcription factor, which was negatively associated with *CCR6* mRNA (*r* = −0.60, *P* = 3.94 × 10^−9^). At the mRNA level, the eQTL effect of *CCR6* was validated (*P* = 4.39 × 10^−2^), and *CCR6* was positively associated with the expression of *CCR4* and *IL-17A* rather than that of *CXCR3* and *IFNG*. At the protein level, a higher CCR6^+^ cell ratio was observed in a risk allele dose-dependent manner in lymphocytes (*P* = 3.57 × 10^−2^), CD3^+^ T cells (*P* = 4.54 × 10^−2^), and CD4^+^ T cells (*P* = 1.32 × 10^−2^), but not in CD8^+^ T cells. Clinical-pathological analysis showed that rs3093023 risk allele was significantly associated with diastolic blood pressure, serum creatinine, and high ratio of tubular atrophy/interstitial fibrosis. Overall, the rs3093023 was prioritized as the function variant in *CCR6*, which may contribute to IgAN susceptibility by regulating Th17 cells.

## Introduction

Immunoglobulin A nephropathy (IgAN) is the most common form of primary glomerulonephritis worldwide and is a major cause of end-stage renal disease in young adults. The variable prevalence among different ethnicities (1) and high familial aggregation (2) suggest that genetic components play an important role in IgAN pathogenesis. However, the known susceptibility loci of IgAN together explains no more than 10% of the disease heritability (3–7), suggesting that the genetic loci remain to be investigated.

Genetic studies have demonstrated the association between C-C motif chemokine receptor 6 gene (*CCR6*) and susceptibility to various immune-related diseases, such as rheumatoid arthritis (8, 9), psoriasis (10), and lupus nephritis (11). CCR6 is the unique receptor for chemokine C-C motif chemokine ligand 20 (CCL20) and is a specific surface marker for interleukin (IL)-17- producing Th17 cells (12). CCR6–CCL20 signaling was reported to play an important role in recruiting Th17 cells to inflammatory sites (13). For example, synoviocytes from mouse and human arthritic joints could produce CCL20 and consequently recruit Th17 cells to the inflamed joints (14). In patients with psoriasis, IL-17-treated epidermal keratinocytes promoted CCL20 production, mediating Th17 cell entry to the lesional skin (15). Furthermore, patients with lupus nephritis have increased levels of Th17 cells (16); in glomerulonephritis, Th17 cells migrate from gut to kidneys *via* CCR6–CCL20 signaling (17).

Recent studies have highlighted the roles of Th17 cells and CCR6–CCL20 signaling in IgAN (18). Th17 cell levels were increased in patients with IgAN compared with healthy controls (19). IgAN mice infected with hemolytic streptococcus showed higher Th17 cell levels in renal lymphocytes, while the addition of CCL20 antibodies could suppress the increase (20). Furthermore, IgA1-stimulated mesangial cells from patients with IgAN produced more CCL20 and attracted CCR6^+^ Th17 cells to kidneys (21). In genetic studies, *CCR6* was suggested to be a shared gene of IgAN (6); however, the functional variants associated with IgAN susceptibility were still unclear.

In this study, we conducted a two-stage genetic association study to identify *CCR6* functional variants associated with IgAN susceptibility, followed by *in silico* regulatory effect prediction, expression association analysis, and clinical-pathological association analysis.

## Participants and Methods

### Participants

**Figure 1:**
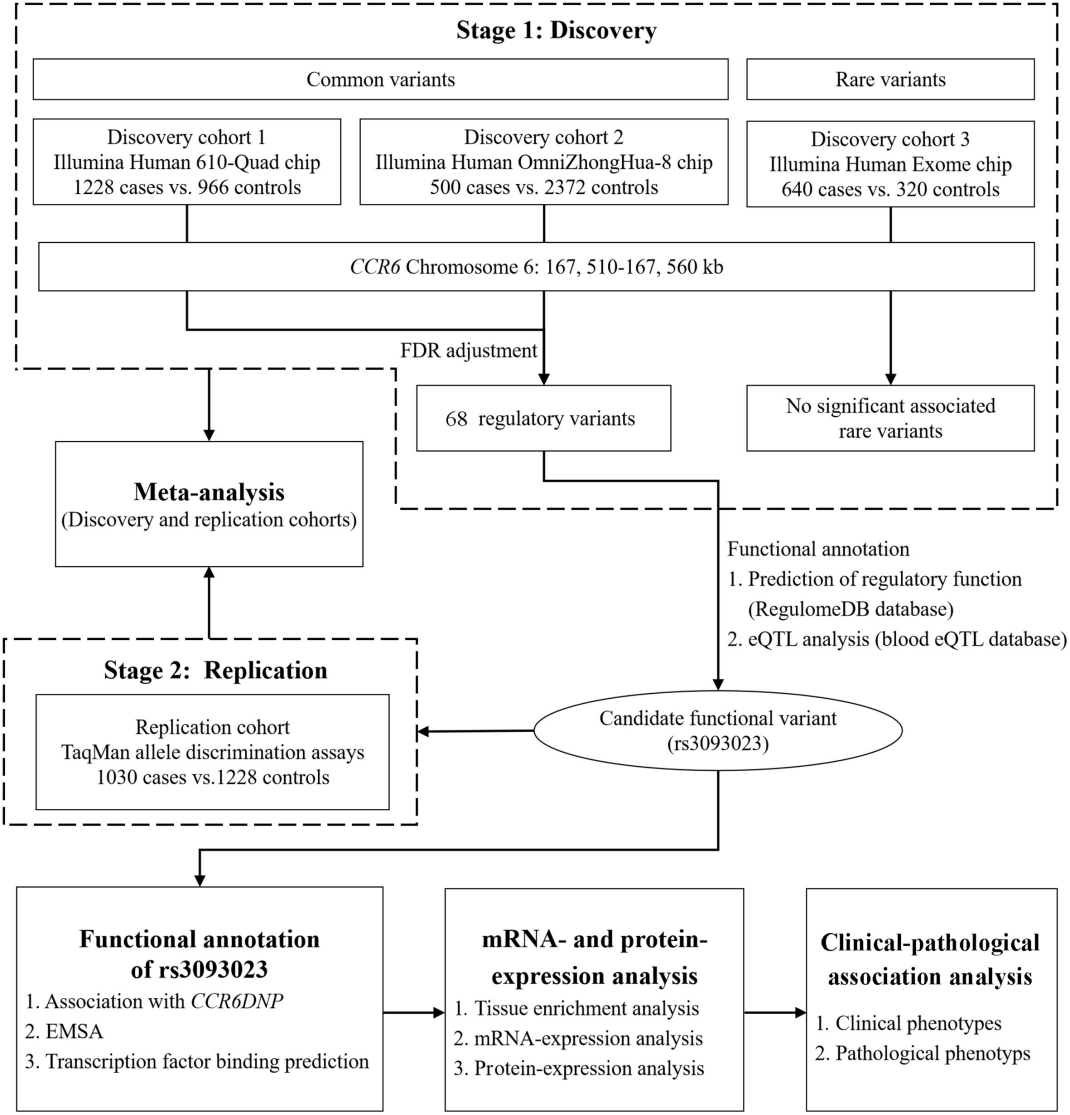
Flowchart of the study design. In the discovery stage, we conducted a genetic association study between the *CCR6* common and rare variants and IgAN using multi-platform chips. The replication stage was aimed at replicating the functional loci discovered in the discovery stage using an independent population. After the two-stage genetic association analysis, we conducted *in silico* functional prediction, expression association analysis, and clinical-pathological association analysis.

In the discovery stage, three independent cohorts were enrolled (Figure 1). Cohort 1 consisted of 1,228 patients with IgAN (age: 31.10 ± 10.70 years, male ratio: 54.00%) and 966 healthy controls (age: 31.54 ± 8.43 years, male ratio: 59.13%), cohort 2 consisted of 500 patients with IgAN (age: 36.70 ± 12.50 years, male ratio: 54.70%) and 2,372 healthy controls (age: 34.81 ± 14.9 years, male ratio: 55.32%), and cohort 3 consisted of 640 patients with IgAN (age: 36.50 ± 13.50 years, male ratio: 48.50%) and 320 healthy controls (age: 36.47 ± 13.45 years, male ratio: 56.56%). For replication, we enrolled 1,030 patients with IgAN (age: 35.91 ± 13.07 years, male ratio: 51.55%) and 1,228 healthy controls (age: 38.6 ± 12.97 years, male ratio: 51.47%). All participants were unrelated northern Han Chinese. The study was approved by the Medical Ethics Committee of Peking University First Hospital (No. [2019]320), and the informed consent was provided by the participants.

All patients with IgAN were diagnosed based on the dominant deposition of IgA in the glomerular mesangium by immunofluorescence, while patients with secondary causes, such as IgA vasculitis, systemic lupus erythematosus, or liver cirrhosis, were excluded. Their baseline demographic and clinical data at renal biopsy, such as age, sex, systolic blood pressure (SBP), diastolic blood pressure (DBP), serum creatinine (Scr), and 24 h proteinuria, were collected. The estimated glomerular filtration rate (eGFR) was calculated using the Chronic Kidney Disease Epidemiology Collaboration equation (22). Pathological characteristics were scored according to the Oxford classification (23).

### Genotyping of the *CCR6* Variants

In the discovery stage, variants in a region spanning 50 kb (chromosome 6: 167, 510–167, 560 kb) encompassing *CCR6* and its downstream and upstream were derived from Illumina chips (USA). More specifically, to analyze the associations of common variants, genotyping information was obtained from the Illumina Human 610-Quad chip (cohort 1) and the Illumina Human OmniZhongHua-8 chip (cohort 2). To analyze the associations of rare variants, genotyping information was obtained from the Illumina Human Exome chip (cohort 3). In the replication stage, the candidate functional variants were genotyped using TaqMan allele discrimination assays (Applied Biosystems, USA).

### *In silico* Functional Annotation

To identify the candidate functional variants, regulatory features were annotated using the RegulomeDB database (24) (http://www.regulomedb.org), and the expression quantitative trait loci (eQTL) were analyzed using the blood eQTL database (25) (http://www.genenetwork.nl/bloodeqtlbrowser/). The potential transcription factor was predicted using the RegulomeDB and JASPAR (26) (http://jaspar.binf.ku.dk/) databases. The regulatory effects of the functional SNP and its proxies, such as enhancer histone marks, promoter histone marks, and DNase hypersensitivity, in different tissues/cells were annotated using the HaploReg database (27) (http://www.broadinstitute.org/mammals/haploreg/haploreg.php).

### Sequencing of *CCR6* Dinucleotide Polymorphism (*CCR6DNP*)

A triallelic dinucleotide polymorphism of *CCR6, CCR6DNP*, was reported to be a disease-causing variation that can affect the *CCR6* expression in rheumatoid arthritis (8). To further investigate the potential functional variant, we determined whether *CCR6DNP* could tag our candidate functional variants. We randomly selected 361 people (166 cases vs. 195 controls) based on the genotypes of the candidate functional variants. Genomic DNA was extracted from whole blood samples and amplified using forward (5′-CAACCACCTTTGAAAGAGCAG-3′) and reverse (5′-CCCTTGTTCATCCCAACCT-3′) primers (8). The DNA products underwent Sanger sequencing, and the signals were analyzed by Chromas (Technelysium Pty, Australia) (Supplementary Figure 1).

### Isolation of Peripheral Blood Mononuclear Cells (PBMCs)

PBMCs were isolated from fresh peripheral blood samples collected from the participants using Ficoll-Paque density gradient centrifugation. Briefly, each sample was centrifuged at 600 g for 30 min to separate plasma. The sample was mixed with phosphate-buffered saline (PBS) in a 1:1 ratio and then slowly transferred onto Ficoll-Paque PLUS (GE Healthcare Bioscience, USA) in a 2:1 ratio. After 30-min centrifugation at 600 g, the upper thin white layer of PBMCs was transferred to a centrifuge tube and resuspended in PBS for further use.

### Electrophoretic Mobility Shift Assay (EMSA)

Nuclear protein was extracted from the PBMCs using the NE-PER Nuclear and Cytoplasmic Extraction Reagent Kit (ThermoFisher Scientific, USA). Biotinylated oligonucleotide probes were designed to correspond to the genomic sequences surrounding the candidate functional variants (8) (Table 1). EMSA was performed using a LightShift Chemiluminescent EMSA Kit (Thermo Scientific, USA) according to the manufacturer's instruction. Briefly, the binding reaction mixtures were set up containing 10 μg nuclear extract, 10× binding buffer (100 mM Tris, 500 mM KCl, 10 mM dithiothreitol), 50% glycerol, 100 mM MgCl_2_, poly(dI:dC) [poly(deoxyinosinic-deoxycytidylic) acid], and 1% Nonidet P-40 of up to 20 μl. The mixtures were incubated on ice for 15 min before the probe was added and were incubated for another 20 min at room temperature. For the competition assay, 1 μl of 100-fold excess amounts of unlabeled cold oligonucleotides against the biotinylated oligonucleotide probes used were incubated with the nuclear extract. Then, the DNA-nuclear protein complexes were run on 5% polyacrylamide gels at 50 V for 30 min and transferred to a nylon membrane at 200 mA for 30 min. After incubating the membrane with an antibody, the membrane was exposed and scanned using an LAS-3000 Imaging System (GE Healthcare Bioscience, USA). The experiment was repeated in triplicate.

**Table 1:** Oligonucleotide probes for EMSA.

**Alleles**	**Sequences**
rs3093023-Forward-A:	5′-CTATGCAAATGAACAATGTGATTTTAAATTT-3′
rs3093023-Reverse-T:	5′-AAATTTAAAATCACATTGTTCATTTGCATAG-3′
rs3093023-Forward-G:	5′-CTATGCAAATGAACAGTGTGATTTTAAATTT-3′
rs3093023-Reverse-C:	5′-AAATTTAAAATCACACTGTTCATTTGCATAG-3′
*CCR6DNP*-Forward-TG:	5′-GTGGCTGCTGCAGAATGGGGGGTGCTGGTGG-3′
*CCR6DNP*-Reverse-CA:	5′-CCACCAGCACCCCCCATTCTGCAGCAGCCAC-3′
*CCR6DNP*-Forward-CG:	5′-GTGGCTGCTGCAGAACGGGGGGTGCTGGTGG-3′
*CCR6DNP*-Reverse-CG:	5′-CCACCAGCACCCCCCGTTCTGCAGCAGCCAC-3′
*CCR6DNP*-Forward-CA:	5′-GTGGCTGCTGCAGAACAGGGGGTGCTGGTGG-3′
*CCR6DNP*-Reverse-TG:	5′-CCACCAGCACCCCCTGTTCTGCAGCAGCCAC-3′

*EMSA, electrophoretic mobility shift assay*.

### Gene Expression

Total RNA from PBMCs were extracted with TRIzol Reagent (Invitrogen, USA) following the manufacturer's instruction. To ensure that the total RNA was free of DNA, the treatment with DNaseI was applied. The quantifications of *CXCR3* (C-X-C motif chemokine receptor 3), *CCR4, CCR6, IL-17A* (interleukin-17A), *INFG* (interferon-γ), *Pou2f1* (POU class 2 homeobox 1), and *Sox_10* (SRY-box transcription factor 10) expression of PBMCs were tested by RNA microarrays, which was performed by Compass Biotechnology Co., Ltd (Beijing, China) using the HumanHT-12 v4.0 Gene Expression BeadChip (Illumina, USA).

### Flow Cytometry

The CCR6 expression of lymphocytes and T-cell subtypes were detected by flow cytometry. Briefly, 500 μl of peripheral blood samples were incubated with antibodies against surface markers (anti-CD3 FITC, anti-CD4 APC, anti-CD8 PerCP-Cy5.5, and anti-CCR6 PE) at 4°C in the dark for 30 min. After erythrocyte lysis, the samples were washed with PBS and acquired by the BD FACSCanto II Flow Cytometry System (BD Biosciences, USA). About 10,000 lymphocytes were measured for each experiment. All manipulations were performed within 2 h after fresh peripheral blood was collected to avoid much death cells. The data were analyzed using FlowJo (Tree Star, USA); Supplementary Figure 2 shows the details of the gating strategy.

### Statistical Analysis

Quality control for the discovery cohorts was performed using PLINK (http://www.cog-genomics.org/plink2/). We excluded samples with (i) sex inconsistencies, (ii) missing genotype call rate > 5%, and (iii) related or cryptically related individuals and single-nucleotide polymorphisms (SNPs) with (i) the missing call rate > 5%, (ii) minor allele frequency <1%, and (iii) deviation from the Hardy–Weinberg equilibrium. For rare variant quality control, minor allele frequency <1% was not applied. Imputation was performed using IMPUTE2. The reference panel comprised data from 1000 Genomes Project Phase 3 haplotypes (Nov 2014, build 37) (28). Supplementary Table 1 shows the details of the quality control and imputation.

Allelic and genotypic associations were assessed using PLINK to yield odds ratio (OR) with 95% confidence interval (95% CI), and *p*-value was adjusted for the false discovery rate method (29). Meta-analysis was performed with STATA (Stata Corp, USA) using the Mantel–Haenszel test, and the pooled OR was calculated using a fixed-effects model. Statistical power was calculated using Power and Sample Size Calculation (http://biostat.mc.vanderbilt.edu/PowerSampleSize). Quantitative variables with normal distribution were expressed as means and standard deviations, and Student's *t*-test was performed. Quantitative variables with non-normal distribution were expressed as medians and interquartile ranges, and Mann–Whitney *U* test was performed. Qualitative variables were summarized as absolute frequencies and percentages, and chi-square test was performed. The association between two continuous variables was determined using Pearson's correlation and linear regression analyses. The results were analyzed using SPSS 22.0 (SPSS Inc., USA). A two-tailed *P* < 0.05 was considered statistically significant.

## Results

### Identification of *CCR6* Functional Variants

After adjusting for the false discovery rate, common variant-based meta-analysis of cohort 1 and cohort 2 identified 68 significantly associated regulatory variants, and rs3093018 was the top associated SNP (OR = 0.85, *P* = 6.88 × 10^−4^) (Supplementary Figure 3, Supplementary Table 2). The rare variant-based association analysis of cohort 3 failed to detect significantly associated rare variants. However, the exome chip replicated the associations of rs3093023 and rs3093024, which were among the 68 regulatory variants (rs3093023, *P* = 0.009; rs3093024, *P* = 0.010) (Supplementary Table 3, Supplementary Figure 4). *In silico* functional annotation showed that eight SNPs (rs3093023, rs1331299, rs975822, rs6907666, rs968333, rs6908364, rs6456158, and rs3093026) were likely to affect transcription factor binding and gene expression, while seven SNPs (rs3093023, rs968334, rs1571878, rs3093024, rs3093025, rs4709148, and rs10946216) showed the eQTL effects (Supplementary Table 4). Accordingly, rs3093023, with both statistical association and the eQTL effect, was prioritized as the candidate functional SNP. Consistently, linkage disequilibrium (LD) analysis showed that the other SNPs with the eQTL effect were in strong LD (*r*^2^ > 0.8) with rs3093023 (Supplementary Table 5).

### Replication and Meta-Analysis

**Figure 2:**
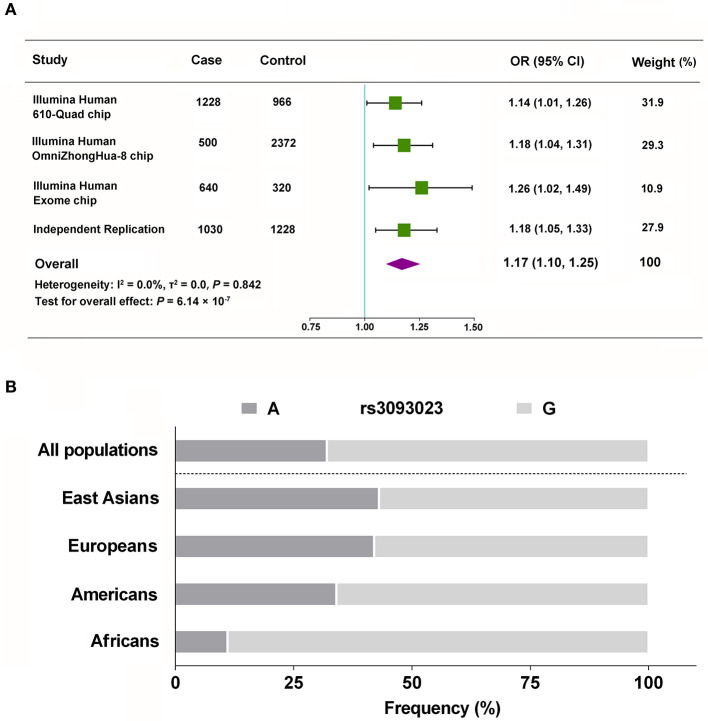
Meta-analysis of rs3093023. **(A)** The combined analysis with the discovery and replication cohorts. **(B)** Racial distribution of the rs3093023 alleles. OR, odds ratio; 95% CI, 95% confidence interval.

We validated the genetic association between rs3093023 and IgAN in an independent replication cohort. rs3093024, which was in almost absolute LD with rs3093023 (*r*^2^ = 0.98), was used as the positive control. rs3093023 was associated with IgAN susceptibility in the allele model (OR = 1.18, 95% CI: 1.05–1.33, *P* = 5.56 × 10^−3^), the co-dominant model (OR = 1.37, 95% CI: 1.08–1.74, *P* = 1.00 × 10^−2^), and the dominant model (OR = 1.26, 95% CI: 1.06–1.51, *P* = 9.22 × 10^−3^) (Table 2). Similar results were observed for rs3093024. The meta-analysis reinforced the genetic association of rs3093023 (OR = 1.17, 95% CI: 1.10–1.25, *P* = 6.14 × 10^−7^) (Figure 2A). Furthermore, the frequency of the rs3093023 A risk allele was high in east Asians, followed by Europeans and Americans; its frequency was lowest in Africans (Figure 2B), which is consistent with the epidemiological trend of IgAN (30).

**Table 2:** Allele and genotype frequencies of rs3093023 and rs3093024 in the replication cohort.

**Genetic model**	**Genotypes**	**Cases *N* (%)**	**Controls *N* (%)**	**OR (95% CI)**	***P*-value**
**Rs3093023**					
Alleles	G	11,36 (55.15)	1,455 (59.24)	Refer	
	A	924 (44.85)	1,001 (40.76)	1.18 (1.05, 1.33)	5.56 × 10^−3^
Co-dominant model	G/G	318 (30.87)	443 (36.07)	Refer	
	A/G	500 (48.55)	569 (46.34)	1.12 (0.89, 1.40)	3.34 × 10^−1^
	A/A	212 (20.58)	216 (17.59)	1.37 (1.08, 1.74)	1.00 × 10^−2^
Dominant model	G/G	318 (30.87)	443 (36.07)	Refer	
	A/A+A/G	712 (69.13)	785 (63.73)	1.26 (1.06, 1.51)	9.22 × 10^−3^
Recessive model	G/G+A/G	818 (79.42)	1,012 (82.41)	Refer	
	A/A	212 (20.58)	216 (17.59)	1.21 (0.98, 1.50)	7.08 × 10^−2^
**Rs3093024**					
Alleles	G	1,131 (54.90)	1,456 (59.28)	Refer	
	A	929 (45.10)	1,000 (40.72)	1.20 (1.06, 1.35)	3.04 × 10^−3^
Co-dominant model	G/G	318 (30.87)	442 (35.99)	Refer	
	A/G	495 (48.06)	572 (46.58)	1.20 (0.99, 1.45)	5.39 × 10^−2^
	A/A	217 (21.07)	214 (17.43)	1.41 (1.11, 1.79)	4.63 × 10^−3^
Dominant model	G/G	318 (30.87)	442 (35.99)	Refer	
	A/A+A/G	712 (69.13)	786 (64.01)	1.26 (1.06, 1.51)	1.04 × 10^−2^
Recessive model	G/G+A/G	813 (78.93)	1,014 (82.57)	Refer	
	A/A	217 (21.07)	214 (17.43)	1.27 (1.03, 1.56)	2.85 × 10^−2^

### Functional Analysis of rs3093023

**Figure 3:**
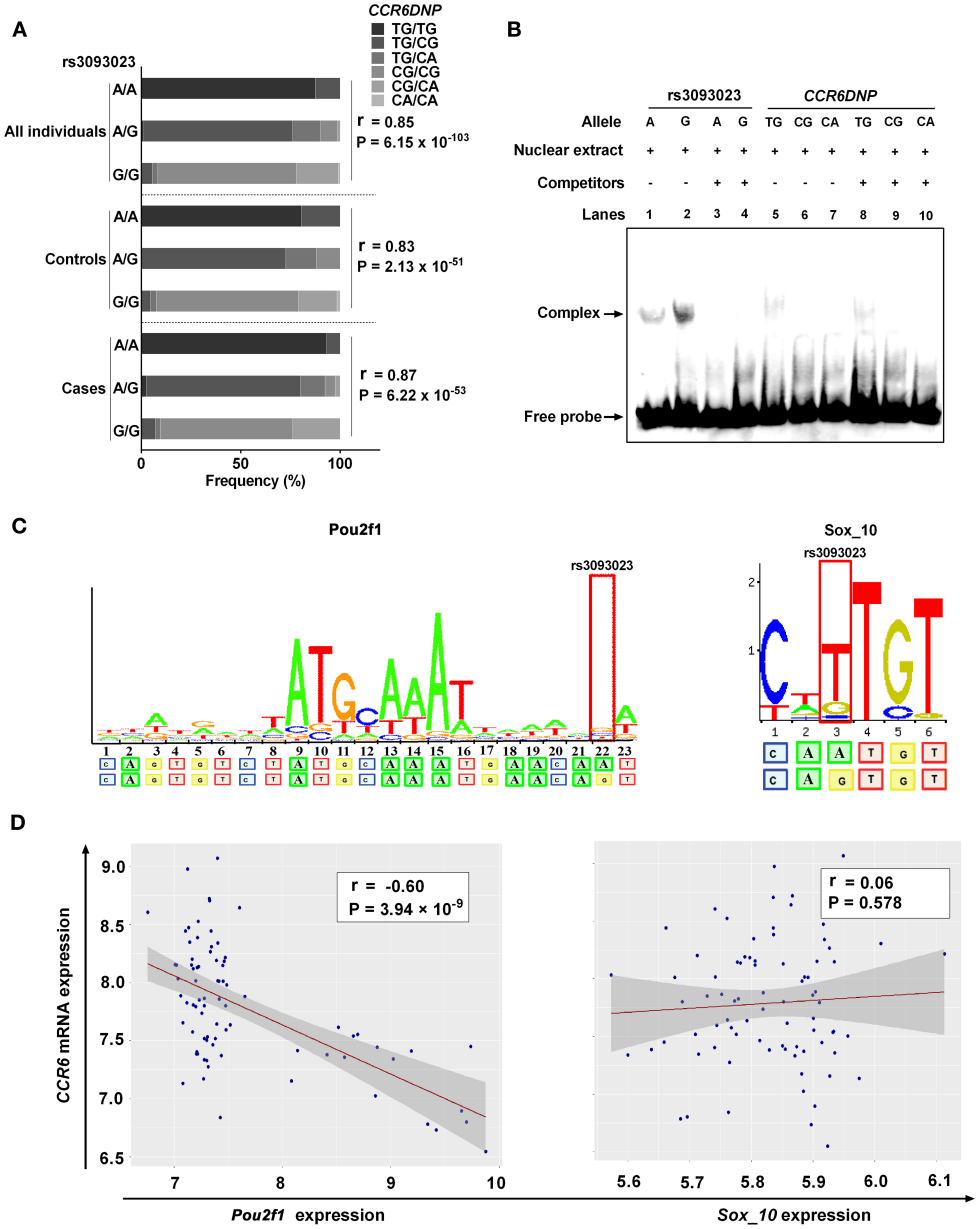
Functional annotation of rs3093023. **(A)** The association between rs3093023 and *CCR6DNP*. **(B)** EMSA. **(C)** Prediction of transcription factors. **(D)** Analysis of expression association. EMSA, electrophoretic mobility shift assay.

The distribution of the *CCR6DNP* genotypes in 361 people was as follows: TG/TG (19.94%), TG/CG (40.17%), TG/CA (7.48%), CG/CG (25.21%), CG/CA (6.92%), and CA/CA (0.28%). The association analysis showed that the rs3093023 A allele could tag the *CCR6DNP* TG allele in the entire genotyped cohort (cases, *r* = 0.87, *P* = 6.22 × 10^−53^; controls, r = 0.83, *P* = 2.13 × 10^−51^; all individuals, *r* = 0.85, *P* = 6.15 × 10^−103^) (Figure 3A, Supplementary Table 6).

We performed EMSA to investigate whether rs3093023 or *CCR6DNP* was the functional variant in IgAN. The oligonucleotide probe containing the rs3093023 allele showed significant mobility shifts, and the protective allele G bound much more protein than the risk allele A (Figure 3B, lanes 1 and 2). Moreover, the excessive unlabeled competitor probes abolished the DNA-nuclear protein complexes (Figure 3B, lanes 3 and 4). However, the oligonucleotide probe containing the *CCR6DNP* allele showed negligible mobility shifts (Figure 3B, lanes 5–7), and the excessive competitor probes did not abolish the DNA-nuclear protein complexes (Figure 3B, lanes 8–10).

In the RegulomeDB and JASPAR databases, we observed a higher affinity of Pou2f1 and Sox_10 for the regulatory motif for the rs3093023 G allele relative to the rs3093023 A allele (Figure 3C). However, the *CCR6* mRNA expression was negatively associated with the *Pou2f1* expression (*r* = −0.60, *P* = 3.94 × 10^−9^) rather than with the *Sox_10* expression (*r* = 0.06, *P* = 0.578) (Figure 3D), suggesting that Pou2f1 may be the key transcription factor regulating the *CCR6* expression *via* rs3093023.

### *CCR6* Expression Is Increased in Individuals With the Risk Genotype

**Figure 4:**
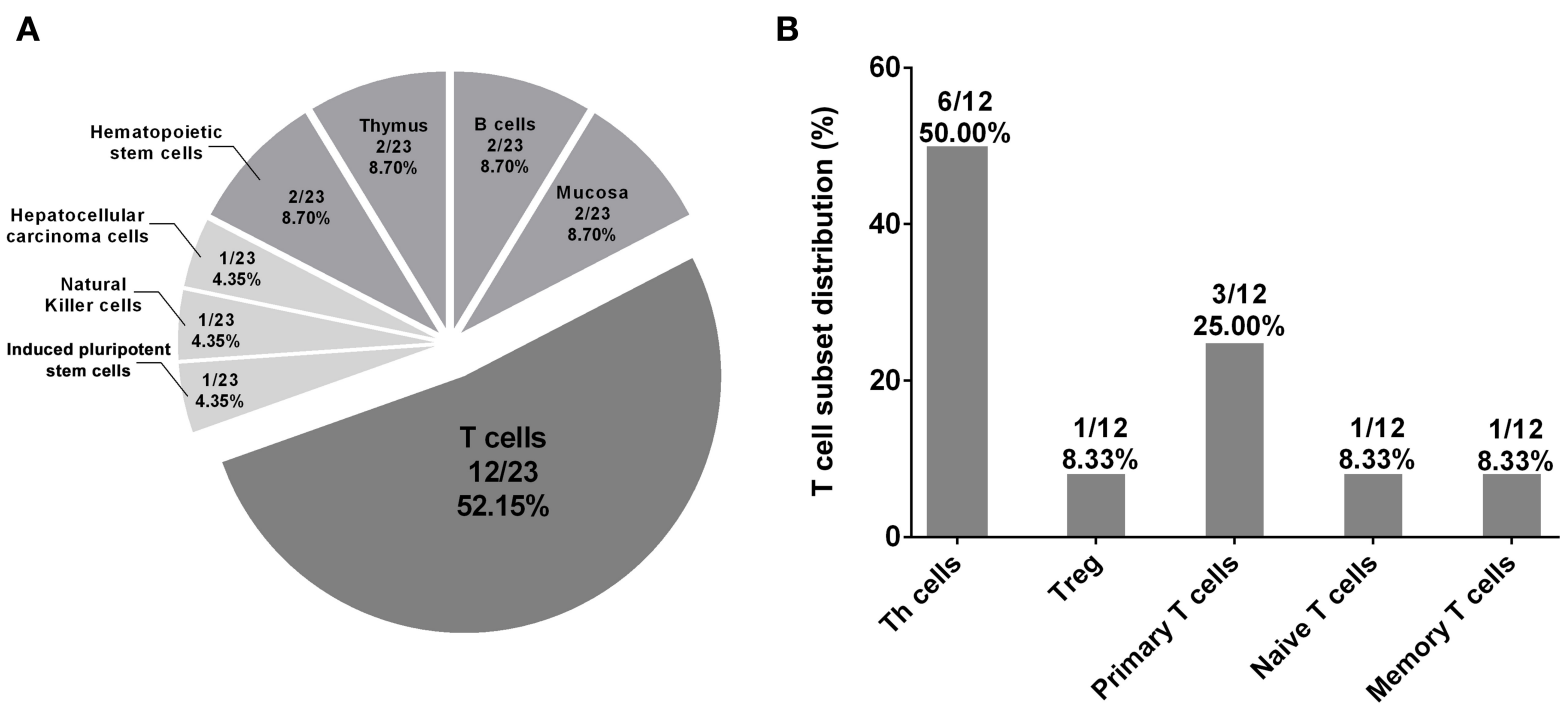
Tissue enrichment analysis of regulatory effects of rs3093023 and its proxies. **(A)** The significantly enriched tissues/cells (*p* < 0.05) of the regulatory effects of rs3093023 and its proxies. **(B)** The distribution of T cell subset enriched for regulatory effects of rs3093023 and its proxies. SNPs, single-nucleotide polymorphisms.

We further used the HaploReg database to annotate the regulatory effects of rs3093023 and its proxy SNPs (rs968334, rs3093025, rs61056617, rs3093024, rs1854853, rs200505068, rs10946216, rs3093019, rs3093018, rs1571878, and rs3093017) in 127 tissues/cells. The results showed that the regulatory effects of rs3093023 and its proxies were significantly enriched in 23 tissues/cells (*p* < 0.05) (Supplementary Table 7). Among the 23 tissues/cells, T cells (12/23, 52.15%), mainly T-helper (Th) cells (6/23, 26.09%), accounted for the most in Figure 4, which suggests that rs3093023 may participate in the pathogenesis of IgAN by regulating T cells.

**Figure 5:**
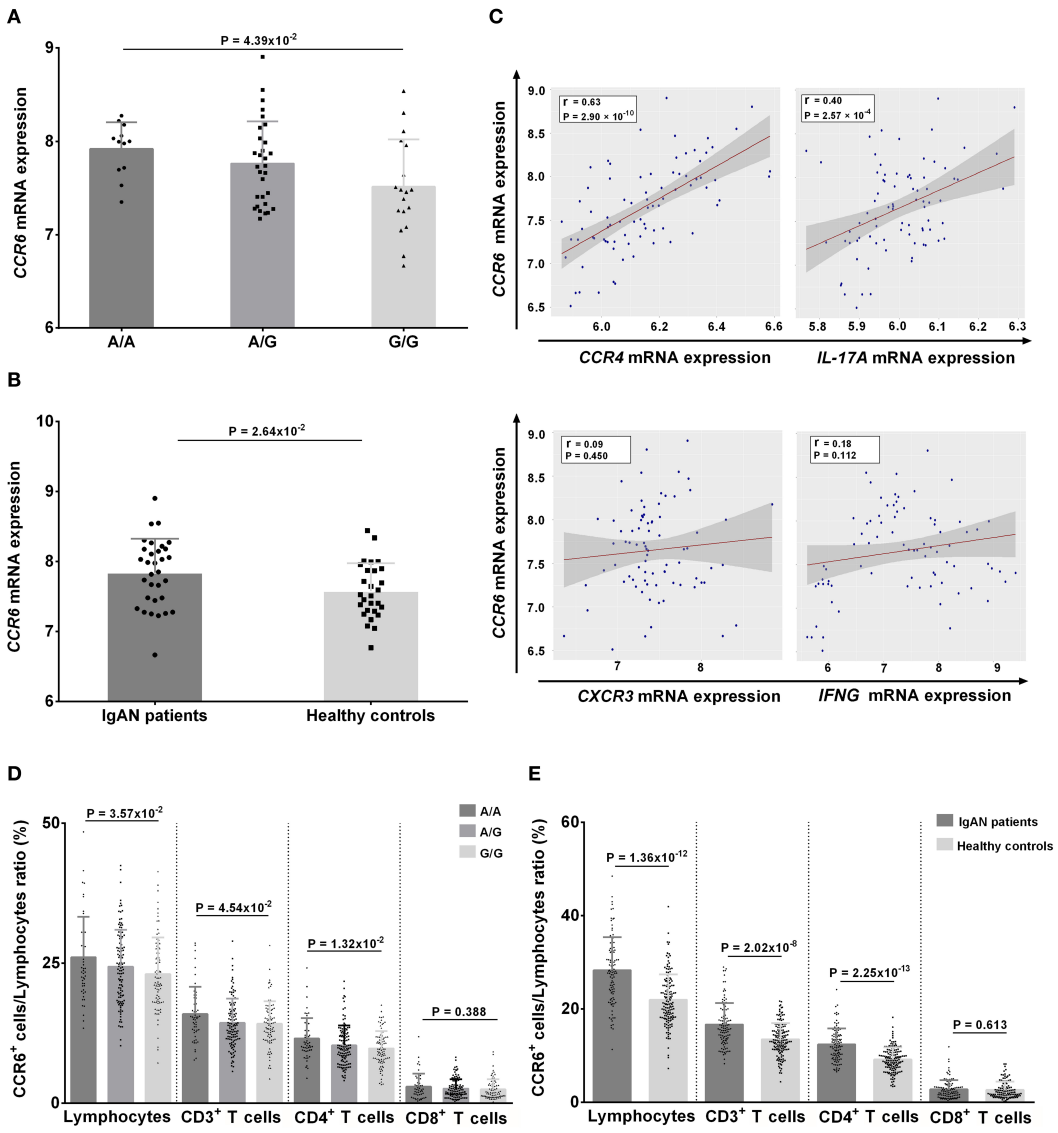
*CCR6* expression in individuals with rs3093023 genotypes. **(A)** The *CCR6* mRNA expression in individuals with different rs3093023 genotypes. **(B)** The *CCR6* mRNA expression in IgAN patients and controls. **(C)** The association between *CCR6* mRNA expression and other cell surface markers of Th1 and Th17 cells. **(D)** The CCR6 protein expression in individuals with different rs3093023 genotypes. **(E)** The CCR6 protein expression in IgAN patients and controls.

At mRNA level, individuals with the risk genotype A/A had significantly higher *CCR6* mRNA levels compared with individuals with the protective genotype G/G, and these levels were dose-dependent on the number of A alleles (A/A vs. A/G vs. G/G: 7.91 ± 0.29 vs. 7.76 ± 0.45 vs. 7.51 ± 0.51, respectively, *P* = 4.39 × 10^−2^) (Figure 5A). The *CCR6* mRNA levels were also higher in patients with IgAN than in controls (7.84 ± 0.48 vs. 7.58 ± 0.40, *P* = 2.64 × 10^−2^) (Figure 5B). Moreover, the *CCR6* mRNA expression was positively associated with the expression of *CCR4* (*r* = 0.63, *P* = 2.9 × 10^−10^) and *IL-17A* (*r* = 0.40, *P* = 2.57 × 10^−4^) but not with the expression of *CXCR3* (*r* = 0.09, *P* = 0.450) and *IFNG* (*r* = 0.18, *P* = 0.112) (Figure 5C), suggesting that rs3093023 may regulate the *CCR6* mRNA expression in Th17 cells.

At the protein level, we analyzed the associations between CCR6^+^ T cell subsets and rs3093023 genotypes. Consistently, higher CCR6^+^ cell ratios were observed in a risk allele dose-dependent manner (A/A > A/G > G/G) in lymphocytes (26.07 ± 7.23 vs. 24.41 ± 6.57 vs. 23.07 ± 6.54, respectively, *P* = 3.57 × 10^−2^), CD3^+^ T cells (15.96 ± 4.84 vs. 14.37 ± 4.34 vs. 14.21 ± 4.04, respectively, *P* = 4.54 × 10^−2^), and CD4^+^ T cells (11.56 ± 3.64 vs. 10.35 ± 3.56 vs. 9.81 ± 3.11, respectively, *P* = 1.32 × 10^−2^), but not in CD8^+^ T cells (2.98 ± 2.32 vs. 2.64 ± 1.69 vs. 2.51 ± 1.88, respectively, *P* = 0.388) (Figure 5D). A higher CCR6^+^ cell ratio was also observed in patients with IgAN for lymphocytes (28.31 ± 7.10 vs. 21.98 ± 5.47, *P* = 1.36 × 10^−12^), CD3^+^ T cells (16.66 ± 4.61 vs. 13.52 ± 3.43, *P* = 2.02 × 10^−8^), and CD4^+^ T cells (12.39 ± 3.46 vs. 9.21 ± 2.58, *P* = 2.25 × 10^−13^) (Figure 5E).

### Association Between rs3093023 Genotypes With IgAN Phenotypes

DBP and Scr were significantly higher in patients with the risk genotype A/A than in patients with the protective genotype G/G (Table 3), and the increases were dose-dependent on the number of risk allele A (80.73 ± 13.04 mmHg vs. 78.85 ± 11.55 mmHg vs. 78.68 ± 11.64 mmHg, respectively, *P* = 0.023; 116.03 ± 83.67 μmol/L vs. 109.50 ± 76.23 μmol/L vs. 101.20 ± 52.02 μmol/L, respectively, *P* = 0.006). Moreover, the incidence of tubular atrophy/interstitial fibrosis increased with the number of risk allele A (47.09% vs. 46.17% vs. 38.03%, *P* = 0.034).

**Table 3:** Association of rs3093023 genotypes with IgAN phenotypes.

	**A/A (*n* = 194)**	**A/G (*n* = 524)**	**G/G (*n* = 373)**	***P*-value**
**Clinical phenotypes**				
Age (years)	33.81 ± 11.66	33.89 ± 12.12	34.39 ± 12.23	0.673
**Sex (%)**				
Male	93 (47.94)	258 (49.24)	178 (47.72)	0.892
Female	101 (52.06)	266 (50.76)	195 (52.28)	
SBP (mmHg)	125.53 ± 17.14	124.52 ± 17.11	123.75 ± 16.34	0.298
DBP (mmHg)	80.73 ± 13.04	78.85 ± 11.55	78.68 ± 11.64	0.023
Scr (μmol/L)	116.03 ± 83.67	109.50 ± 76.23	101.20 ± 52.02	0.006
eGFR (mL/min/1.73 m^2^)	80.62 ± 31.50	84.31 ± 32.28	85.61 ± 29.87	0.062
Proteinuria (g/24 h)	2.31 ± 2.55	2.23 ± 2.34	2.15 ± 2.24	0.611
**Pathological phenotypes**				
M0/M1 (%)	25/164 (13.23/86.77)	56/453 (11/89)	58/297 (16.34/83.66)	0.074
E0/E1 (%)	123/66 (65.08/34.92)	357/152 (70.14/29.86)	256/99 (72.11/27.89)	0.231
S0/S1 (%)	96/93 (50.79/49.21)	285/224 (55.99/44.01)	208/147 (58.59/41.41)	0.218
T0/T1/T2 (%)	100/60/29 (52.91/31.75/15.34)	274/160/75 (53.83/31.43/14.73)	220/105/30 (61.97/29.58/8.45)	0.027
T0/T(1+2) (%)	100/89 (52.91/47.09)	274/135 (53.83/46.17)	220/135 (61.97/38.03)	0.034

*IgAN, IgA nephropathy; SBP, systolic blood pressure; DBP, diastolic blood pressure; Scr, serum creatinine; eGFR, estimated glomerular filtration rate; M, Mesangial hypercellularity; E, Endocapillary proliferation; S, Segmental sclerosis; T, interstitial fibrosis/tubular atrophy*.

## Discussion

In the discovery stage, we detected 68 common variants in *CCR6* associated with IgAN susceptibility. rs3093023 showed both statistical significance and functional evidence, which was independently replicated. EMSA showed differential affinity of nuclear proteins for the rs3093023 alleles (G > A). *In silico* analysis identified Pou2f1 as a potential transcription factor, which was negatively associated with the *CCR6* expression. Moreover, CCR6 transcript- and protein- expression levels increased with the number of risk allele A, and the *CCR6* mRNA expression was positively associated with the expression of *CCR4* and *IL-17A* rather than with the expression of *CXCR3* and *INFG*. The clinical–pathological analysis showed that DBP, Scr, and incidence of tubular atrophy/interstitial fibrosis increased in a risk allele dose-dependent manner. Taken together, the results suggest that *CCR6* rs3093023 may be involved in IgAN susceptibility by regulating Th17 cells.

*CCR6* is a shared susceptibility gene between IgAN and other immune-related diseases (6). However, the associated *CCR6* variants have not been reported in IgAN. A rheumatoid arthritis GWAS in Japanese identified *CCR6* rs3093024 as the causal variant associated with the *CCR6* expression (6). rs3093023 A causes disease susceptibility in patients with systemic sclerosis (9). Furthermore, the functional *CCR6DNP* affects *CCR6* transcription in rheumatoid arthritis, systemic sclerosis, and Crohn's diseases (6, 8, 9). In this study, we first investigated the associations between IgAN and the *CCR6* common and rare variants. Consistently, most of the *CCR6* susceptibility variants associated with complex immune-related diseases are in the non-coding regions (31). A common variant, rs3093023, was prioritized as a functional variant. Although rs3093023 was in strong LD with the reported functional variant *CCR6DNP* (6), the allelic differences in nuclear protein binding were only observed for rs3093023, suggesting that rs3093023 may be the functional variant involved in IgAN.

Furthermore, our expression analysis indicated that rs3093023 and its proxy SNPs mainly alters the *CCR6* expression in Th cells. Accumulating evidence indicates that changes in CD4^+^ T cells, including T regulatory (Treg) and effector Th cells, are involved in IgAN pathogenesis (18). Th2 polarization and Th17- and Tfh (T follicular helper)-derived interleukin release may promote aberrant IgA1 galactosylation in IgAN (32, 33). CCR6 is a surface marker for IL-17-producing Th17 cells (12) and IFNγ-producing Th1 cells (34). The expression of CCR6 and CCR4 together can identify Th17 cells, whereas the expression of CCR6 and CXCR3 identifies Th1 cells (34). In the present study, CCR6 transcript and protein expression levels increased with the number of risk allele. More importantly, *CCR6* mRNA was positively associated with the expression of *CCR4* and *IL-17A* rather than with the expression of *CXCR3* and *IFNG*. Moreover, the significant association between rs3093023 genotypes and CCR6^+^ cells was mainly observed in CD4^+^ T cells. The results indicate that rs3093023 likely modulates the CCR6 expression in Th17 cells. Increased circulatory Th17 cells have been found in patients with IgAN and a murine model of IgAN (19, 35). The levels of the cytokines secreted by Th17 cells, including CCL20, IL-17A, IL-6, and IL-21, were also increased in kidneys of mice with IgAN (20). IgA1 could promote CCL20 production in mesangial cells from patients with IgAN to attract inflammatory CCR6^+^ Th17 cells to kidneys to accelerate renal injury (21). Notably, B cells have high amounts of CCR6 mRNA and protein (36). CCR6 and the corresponding ligand CCL20 can recruit B cells to renal inflammatory sites (37). However, the present study mainly focused on T cells with the most enrichment of rs3093023, and future studies can evaluate the role of B cells. Taken together, our findings provide further evidence from the genetic point of view that Th17 cells are involved in the pathogenesis of IgAN.

Finally, we observed that DBP, Scr, and the incidence of tubular atrophy/interstitial fibrosis were increased in a risk allele dose-dependent manner, indicating that the functional variant rs3093023 may be associated with IgAN severity. Higher Th17 cell numbers promote IL-17 production, inducing ongoing inflammatory response and renal injury (38). IL-17A activates human proximal renal tubular cells, which subsequently release high amounts of IL-6 and IL-8 (39). In the murine models of nephrotoxic nephritis (40) and ischemia/reperfusion (41), IL-17 deficiency ameliorated glomerular crescent formation and tubulointerstitial injury. In IgAN, serum levels of IL-17A secreted from Th17 cells were increased, and were positively associated with proteinuria (19). Immunohistochemistry has shown that IL-17A is expressed at the renal tubule site in most patients with IgAN (19). Compared with patients without IL-17A expression, patients with IL-17A expression have higher Scr, increased proteinuria, and more severe tubulointerstitial damage (19), which are strongly associated with progression to renal failure (42).

Overall, rs3093023 was prioritized as the functional variant in *CCR6*, which may contribute to IgAN susceptibility by regulating Th17 cells, suggesting that target Th17 cells may be a promising treatment for IgAN. Intriguingly, therapies that target Th17-related cytokines, Th17 intracellular signaling pathways, and Th17-specific transcription factors are available and have been reported to be efficient in the treatment of several immune-related diseases. For example, IL-17 neutralizing monoclonal antibodies, such as secukinumab, ixekizumab, and brodalumab, have been approved for treating psoriasis, psoriatic arthritis, and ankylosing spondylitis (43–47). A phase I randomized controlled trial revealed the potential benefit of GSK2981278, a RORγt inverse agonist, which significantly inhibits the production of Th17-producing cytokines in psoriasis (48). Although there were no studies reporting the efficacy of Th17 blockade in IgAN, two recent cases of IgAN concurrent with psoriasis demonstrated a decrease in proteinuria after the treatment of secukinumab, implying the potential beneficial effect of Th17 blockade in IgAN (49, 50). Collectively, further clinical trials are needed to verify the efficacy of targeting Th17 cells in the treatment of IgAN.

Our study has several limitations. First, as the genetic association was based on a northern Han Chinese population, multiracial replication studies should be conducted in the future. Second, we failed to identify rare variants in *CCR6*, and the effect sizes of the susceptibility locus accounted for <10% disease risk; therefore, additional genetic and environmental risk factors should be investigated. Third, the regulation effects of rs3093023 were mainly derived from *in silico* predictions, more reliable experiments especially in Th17 cells are needed for validation in the future.

In conclusion, through a two-stage genetic association study, we found that the *CCR6* functional variant rs3093023 is involved in IgAN susceptibility *via* the regulation of Th17 cells. Our study also provides genetic evidence that Th17 cells are involved in IgAN and may be useful for understanding the pathogenesis and treatment of the disease.

## Data Availability Statement

The original contributions presented in the study are included in the article/Supplementary Material, further inquiries can be directed to the corresponding author/s.

## Ethics Statement

The studies involving human participants were reviewed and approved by Medical Ethics Committee of Peking University First Hospital (No. [2019]320). The patients/participants provided their written informed consent to participate in this study.

## Author Contributions

YZ, XZ, and HZ conceived the study, generated the original hypothesis, and designed the experiments. YZ performed the experiments. YZ and XL conducted data analysis and drafted the manuscript. YZ, XL, XZ, LL, SS, PH, JL, and HZ recruited and prepared the samples. YZ, and HZ provided financial support. All authors reviewed/edited the manuscript and approved the final version.

## Conflict of Interest

The authors declare that the research was conducted in the absence of any commercial or financial relationships that could be construed as a potential conflict of interest.

## Supplementary Material

Supplementary Figure 1 Genotyping of *CCR6DNP*.

Supplementary Figure 2 Flow cytometry gating strategy for CCR6^+^ cells.

Supplementary Figure 3 Genetic association of *CCR6* common variants and IgAN. IgAN, immunoglobulin A nephropathy.

Supplementary Figure 4 Genetic association of *CCR6* rare variants and IgAN.

Supplementary Table 1 Details of genotyping, quality control, and imputation for each cohort.

Supplementary Table 2 Associated regulatory variants after false discovery rate adjustment in cohort 1 and cohort 2.

Supplementary Table 3 Association of IgAN susceptibility and *CCR6* rare variants in cohort 3. IgAN, immunoglobulin A nephropathy.

Supplementary Table 4 Functional annotations of 68 associated regulatory variants.

Supplementary Table 5 LD analysis of rs3093023 and other *CCR6* SNPs with eQTL effects. eQLT, expression quantitative trait loci.

Supplementary Table 6 Association analysis of rs3093023 and *CCR6DNP*.

Supplementary Table 7 Tissues/cells enrichment for regulatory effects of rs3093023 and its proxy SNPs. SNPs, single-nucleotide polymorphisms.

## Abbreviations

CCL20: C-C motif chemokine ligand 20
CCR4: C-C chemokine receptor 4
CCR6: C-C chemokine receptor 6
CCR6DNP: CCR6 dinucleotide polymorphism
CXCR3: C-X-C motif chemokine receptor 3
DBP: diastolic blood pressure
eGFR: estimated glomerular filtration rate
EMSA: electrophoretic mobility shift assay
eQTL: expression quantitative trait loci
GWAS: genome-wide association study
IgAN: IgA nephropathy
IL-17A: interleukin-17A
INFG: interferon-γ
LD: linkage disequilibrium
OR: odds ratio
PBMCs: peripheral blood mononuclear cells
PBS: phosphate-buffered saline
Pou2f1: POU class 2 homeobox 1
SBP: systolic blood pressure
Scr: serum creatinine
Sox_10: SRY-box transcription factor 10
Tfh: T follicular helper
Th: T helper.
