# Supplementary figures and images for: A Functional Variant rs3093023 in CCR6 Is Associated With IgA Nephropathy by Regulating Th17 Cells in a North Han Chinese Population
Source: Front Immunol. 2021 Feb 25;12:600598. doi: 10.3389/fimmu.2021.600598 (PMC7946973; doi:10.3389/fimmu.2021.600598)

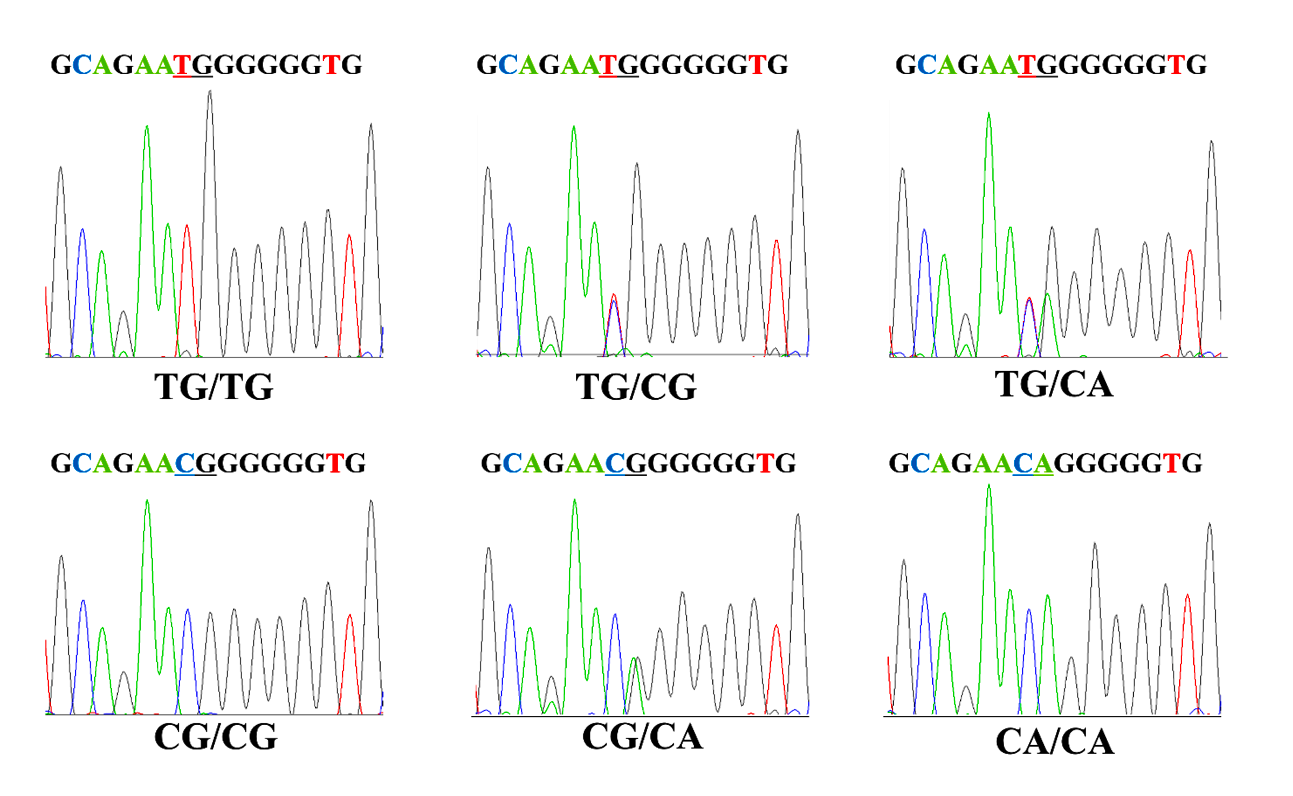

Supplement: Supplementary Figure 1 — Genotyping of CCR6DNP. [file Image_1.TIF]

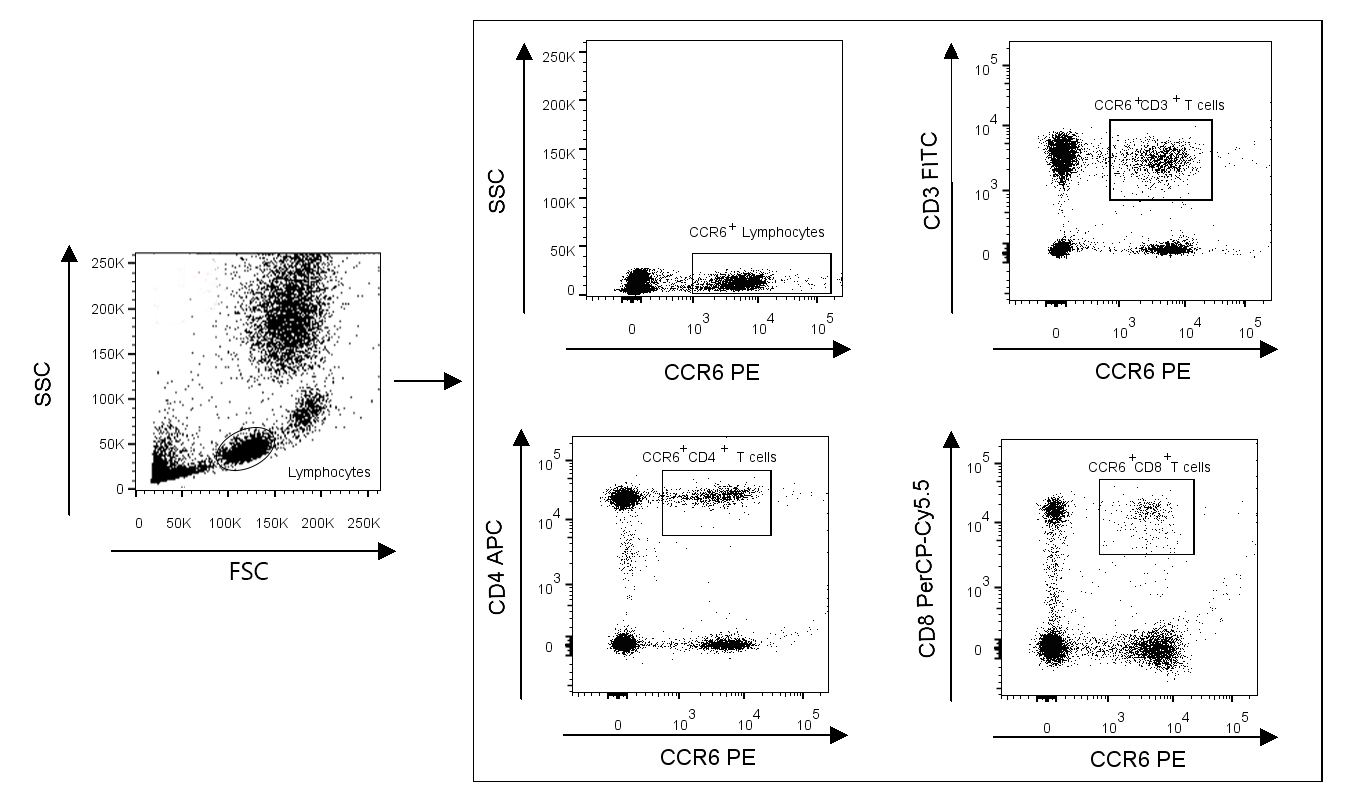

Supplement: Supplementary Figure 2 — Flow cytometry gating strategy for CCR6+ cells. [file Image_2.TIF]

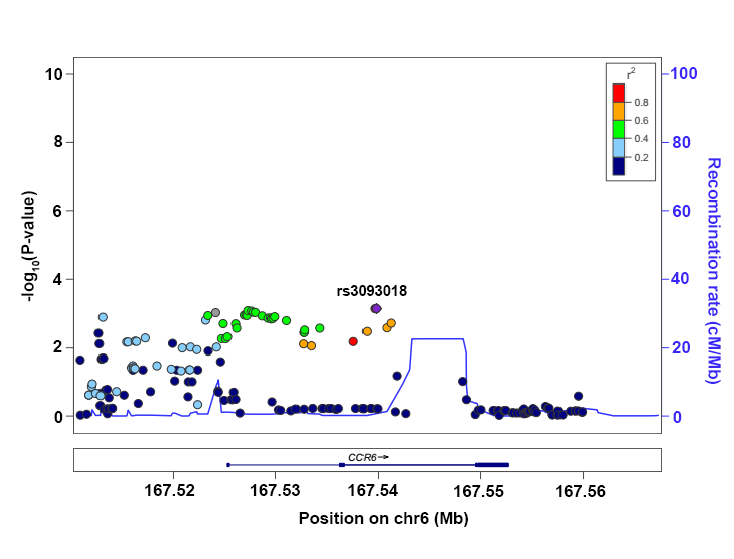

Supplement: Supplementary Figure 3 — Genetic association of CCR6 common variants and IgAN. IgAN, immunoglobulin A nephropathy. [file Image_3.TIF]

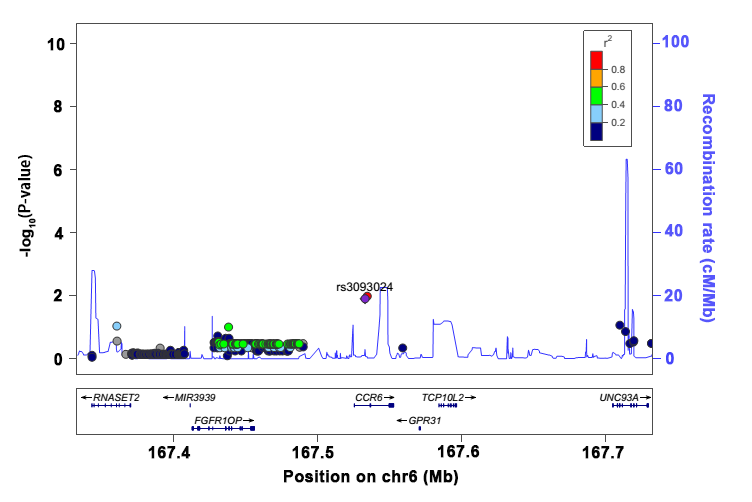

Supplement: Supplementary Figure 4 — Genetic association of CCR6 rare variants and IgAN. [file Image_4.TIF]
